# Is There an Association Between Poor Sleep and Worsened Mental Health Outcomes in Medical Students: A Systematic Review

**DOI:** 10.1192/bjo.2026.11297

**Published:** 2026-06-30

**Authors:** Isabelle Merrony

**Affiliations:** University of Liverpool, Liverpool, United Kingdom

## Abstract

**Aims:**

This review aimed to examine the relationship between sleep disturbances and mental health outcomes in medical students, focusing on key mental health issues such as depression, anxiety, suicidal ideation and dropout intentions. It sought to determine the prevalence of sleep disturbances, assess the strength of the associations with mental health issues and evaluate the quality of the evidence across different study designs using a thematic approach. The study aimed to uncover gaps in the literature, particularly regarding longitudinal research, objective biological sleep assessments and interventional studies.

**Methods:**

Following PRISMA guidelines, a comprehensive search was conducted in Scopus, PubMed and Web of Science, as well as hand-searching and citation tracking. Studies examining sleep disturbances were measured using validated tools such as Pittsburgh Sleep Quality Index (PSQI) and Epworth Sleepiness Scale (ESS). Mental health outcomes such as depression, anxiety and burnout in medical students were included.

An initial appraisal was conducted extracting strengths and weaknesses of each study and then the Joanna Briggs Institute (JBI) Critical Appraisal Tool was used to assessmethodological quality of the included studies. Thematic analysis was applied to compare findings across studies.

**Results:**

Seven studies met the inclusion criteria, comprising a meta-analysis, a longitudinal study, a mixed methods study and four cross-sectional studies. Thematic analysis highlighted four key areas: (a) Prevalence and impact of poor sleep quality; (b) Sleep and mental health; (c) Risk factors for poor sleep and mental health; (d) Coping strategies and sleep’s role.

55% of participants in the Jahrami study reported poor sleep, confirming that there is a high rate among medical students. Poor sleep was linked to depression, anxiety, and suicidal ideation. However, most studies were cross-sectional and used self-reported data which limits causal conclusions. Only one study, Vollmer-Conna explored physiological mechanisms, highlighting the potential need for biological research on sleep and mental health. No study tested interventions aimed at improving sleep and mental health outcomes, leaving a significant gap in the literature for interventional studies.

**Conclusion:**

This review confirms a strong link between poor sleep and worsened mental health in medical students, highlighting the need for interventions within this population. However, longitudinal studies, biological assessments and intervention experiments are needed to establish causality, increase reliability and implement potential interventions to improve the overall wellbeing and performance of medical students who are the future of the healthcare workforce. Medical schools should prioritise sleep hygiene, education, workload adjustments and mental health support to improve student wellbeing throughout the course and prepare them for a career as a doctor.

